# DNA methylation profiles of elderly individuals subjected to indentured childhood labor and trauma

**DOI:** 10.1186/s12881-017-0370-2

**Published:** 2017-02-27

**Authors:** Zoya Marinova, Andreas Maercker, Andreas Küffer, Mark D. Robinson, Tomasz K. Wojdacz, Susanne Walitza, Edna Grünblatt, Andrea Burri

**Affiliations:** 1 Department of Child and Adolescent Psychiatry and Psychotherapy, Psychiatric Hospital, University of Zurich, Neumunsterallee 9, 8032 Zurich, Switzerland; 2 Department of Psychology, University of Zurich, Zurich, Switzerland; 3 University Research Priority Program Dynamics of Healthy Aging, University of Zurich, Zurich, Switzerland; 4 Institute of Molecular Life Sciences, University of Zurich, Zurich, Switzerland; 5 SIB Swiss Institute of Bioinformatics, University of Zurich, Zurich, Switzerland; 6 Aarhus Institute of Advanced Studies, University of Aarhus, Aarhus, Denmark; 7 Neuroscience Center Zurich, University of Zurich and the ETH Zurich, Zurich, Switzerland; 8 Zurich Center for Integrative Human Physiology, University of Zurich, Zurich, Switzerland; 9 Health and Rehabilitation Research Institute, Auckland University of Technology, Auckland, New Zealand; 10 Waitemata Pain Service, Department of Anaesthesia and Perioperative Medicine, North Shore Hospital, Auckland, New Zealand

## Abstract

**Background:**

Childhood trauma is associated with increased vulnerability to mental and somatic disorders later in life. Epigenetic modifications such as DNA methylation are one potential mechanism through which such long-lasting impairments/consequences can be explained. The aim of the present study was to investigate whether childhood trauma is associated with long-term DNA methylation alterations in old age.

**Methods:**

We assessed genome-wide DNA methylation profiles in a cohort of former indentured child laborers (“Verdingkinder”) who suffered severe childhood adversities (*N* = 30; M age = 75.9 years), and compared them to control group with similar demographic characteristics (*N* = 15, M age = 72.8 years). DNA was isolated from epithelial buccal cells and hybridized to the Illumina Infinium 450 k DNA methylation array, which provides coverage of 485,000 methylation sites.

**Results:**

After accounting for batch effects, age, gender and multiple testing, 71 differentially methylated CpG positions were identified between the two groups. They were annotated among others to genes involved in neuronal projections and neuronal development. Some of the identified genes with differential methylation (DLG associated protein 2, mechanistic target of rapamycin) have previously been associated with traumatic stress.

**Conclusions:**

The results indicate specific epigenetic alterations in elderly individuals who were subjected to childhood adversities. Psychiatric and somatic comorbidities as well as differences in buccal epithelial cells proportion may contribute to the observed epigenetic differences.

**Electronic supplementary material:**

The online version of this article (doi:10.1186/s12881-017-0370-2) contains supplementary material, which is available to authorized users.

## Background

Childhood adversities are highly prevalent [1, 2]. In the World Mental Health surveys, prevalence of childhood maltreatment - one type of childhood adversity - has been estimated at 8% for physical abuse, 4.4% for neglect, and 1.6% for sexual abuse [2]. Exposure to childhood adversities is associated with increased vulnerability to a number of mental disorders in adulthood, including major depression, anxiety, suicidal ideation, and posttraumatic stress disorder (PTSD) [3–5]. In addition, childhood trauma has been linked to higher incidence of general medical conditions and increased disability [3]. While it has been consistently demonstrated that childhood adversities are associated with long-term health consequences, the molecular mechanisms through which this occurs remain unknown. It has been hypothesized that a combination of genetic predisposition, epigenetic modifications, and changes in immune and hormonal parameters may be the mechanisms through which childhood trauma can lead to an increased risk of morbidity later in life [6].

Epigenetic modifications include, among others, DNA methylation [7]. So far only few studies have explored DNA methylation differences between victims of childhood trauma and controls on the whole genome level [8–13]. In an attempt to do so, Yang et al. identified differential methylation in 2868 CpG sites in DNA from saliva of maltreated children (*n* = 96) and demographically matched controls (*n* = 96) [11]. Genes relevant to biological processes such as neurogenesis and axonal guidance, as well as a number of disease biomarkers differed in their DNA methylation. In another study, Suderman et al. assessed whole blood DNA methylation profiles of 45 years old individuals subjected to childhood abuse (*n* = 12) and compared these to the methylation profiles of healthy controls (*n* = 28) [10]. Here, the authors found DNA methylation alterations in 997 promoters, mostly related to developmental and regulational functions, and enrichment in genes associated with the WNT signaling pathway. Yet another study looking at children who were institutionalized at birth (*n* = 14) and comparing them to non-institutionalized controls (*n* = 14), found differential methylation in 914 CpG sites located in genes involved in cell signaling, inflammation, but also in neuronal development and communication [12]. Some studies, however, failed to detect significant DNA methylation differences, such as the one conducted by Smith and colleagues who explored peripheral blood DNA methylation profiles in 110 African American individuals, stratified according to childhood trauma and PTSD diagnosis. In that investigation, no significant epigenome-wide differences could be found as a result of childhood abuse. However, CpG sites in five genes, mainly related to immune dysregulation, were differentially methylated in subjects suffering from PTSD in comparison to subjects without PTSD [13].

Overall, previous research has provided evidence for differentially methylated regions associated with previous traumatization and potentially explaining later morbidity. However, most of these studies have been carried out in children or young/middle aged adults, although there is evidence, that long-term epigenetic alterations can also be observed in older individuals. In a study conducted on elderly Dutch individuals subjected to another type of early-life stress (prenatal famine) during World War II and comparing them to their siblings, Tobi et al. found evidence for differential DNA methylation extending to regulatory regions, as well pathways related to growth and metabolism [14]. In contrast, in an investigation of elderly Finnish individuals who experienced separation from their families during the Second World War (*n* = 83) and demographically matched controls (*n* = 83), experiment-wide differences in blood DNA methylation profiles were found only in association with depressive symptoms and not with early life separation [15].

To further investigate the long-term epigenetic changes after childhood traumatization that might explain the higher risk for morbidity in later life, we assessed genome-wide DNA methylation profiles in a group of elderly, previously indentured Swiss child laborers (i.e., “Verdingkinder”) and compared them to a group of controls, consisting of individuals with similar age and gender ratio. The “Verdingkinder” were children who up until the 1950s were removed from their biological families due to different reasons (for example poverty, being born out of wedlock or being orphaned). They were sent to live and work with farmers and many of these children experienced physical, emotional and/or sexual abuse during the period of indentured labor [16, 17]. Therefore, they represent an ideal cohort to be studied in terms of long-term consequences of early childhood trauma.

## Methods

### Study group

The “case” sample of former indentured child laborers belonged to a larger study group from a longitudinal project, aiming at evaluating a range of physical and mental health parameters in traumatized individuals [18–20]. For DNA methylation comparison, a control group with similar demographic characteristics was additionally recruited. The study was approved by the Cantonal Ethic Commission of Zurich – study number KEK-ZH-Nr. 2012–0245. Informed consent was obtained by all participants in the study. Inclusion criteria for both the former indentured child labor group and the control group were: at least 65 years of age, voluntary participation, (Swiss) German speaking, upbringing in rural areas of Switzerland. Specific inclusion criteria for the former indentured child labor group further included: at least one period of indentured child labor. Specific inclusion criteria for the control group were: no indentured child labor or adoptive care, no major childhood trauma.

### Assessment of childhood trauma

Former child laborers were asked about their age at the beginning and end of the indentured child labor experience, as well as the number of different families in which they were placed. To evaluate the presence and extent of childhood trauma, the childhood trauma questionnaire – short form (CTQ-SF) was used in both child laborers and the controls [21, 22]. The CTQ-SF is a retrospective self-report 28-item questionnaire (consisting of 25 clinical items and 3 validity items). Subscales scores for emotional abuse, physical abuse, sexual abuse, emotional neglect and physical neglect are included, each consisting of 5 items with severity scores ranging from 5 to 25. In addition, a summary score (ranging from 25 to 125) for total severity of childhood abuse and neglect can be computed by summing up all 5 subscales. The CTQ-SF has proven to be a reliable measure of childhood abuse and neglect in adult samples in a number of validation studies. A recent validation study of a German version of the CTQ-SF conducted in a sample of Swiss patients and non-clinical participants [23] confirmed the original five-factor model of the original English version and further found high internal consistencies for all scales (physical abuse =0.83, emotional abuse =0.83, sexual abuse =0.9, emotional neglect =0.91), apart from physical neglect (α = 0.53).

### DNA methylation array

Epithelial buccal cells were sampled using Isohelix DNA Buccal Swabs, which have been shown to yield a relatively homogenous population of squamous epithelial cells, with a smaller proportion of blood cells [24]. DNA on the swab heads was stabilized with Isohelix Dri-Capsules (Cell Projects Ltd, Kent, United Kingdom). None of the individuals from whom buccal swabs were collected showed any signs of oral infection, which could lead to higher cellular heterogeneity. Buccal swabs were sent to the “Barts and The London Genome Centre” in the United Kingdom for further processing. DNA was isolated from the buccal swabs using the Isohelix buccal DNA isolation kit (Cell Projects Ltd) and purified with the Zymo ZR-96 DNA clean-up kit (Zymo Research Corporation, Irvine, United States). Before and after purification, DNA concentration was measured with the Qubit 2.0 Fluorometer (Life Technologies). DNA integrity was estimated with agarose gel electrophoresis. After DNA bisulfite conversion using the EZ DNA Methylation kit (Zymo Research Corporation), DNA concentration was again controlled for using Nanodrop (Thermo Scientific). Bisulfite converted DNA was hybridized to the Illumina Infinium 450 k DNA methylation array providing coverage of 485,000 methylation sites for each analyzed sample [25]. The array’s coverage extends to 99% of RefSeq genes, including multiple gene probes, 96% of CpG islands, CpG island shores, and additional content [26].

### DNA methylation data analysis

Raw intensity data files (IDAT files) served as an input and were analyzed in the R environment (http://cran.r-project.org) [27]. Data import, quality control and normalization steps of the *minfi* Bioconductor package were used [28, 29]. Strict quality criteria were introduced in order to decrease the variability of the data set and to allow more reliable detection of true intergroup differences. Samples with fraction of failed probes per sample > 0.05 (one sample) or samples, which in spot-checks of mean fluorescence showed values above a threshold of 0.01 (7 samples), were excluded from the analysis. Probes exhibiting detection *p*-values > 0.01 in one or more samples were excluded (*n* = 451). Background correction and normalization were carried out with the Illumina method implemented in *minfi* (bg.correct = TRUE, normalize = “controls”). The probes on the Illumina Infinium 450 k array measuring single nucleotide polymorphisms (SNPs) (*n* = 65) or non-CpG methylation (*n* = 3026) were filtered out. Probes containing SNPs at the target CpG site or at single base extension position were excluded and SNPs with any minor allele frequency were considered (*n* = 17534). The SNP annotation integrated in the *minfi* package was used [30]. Finally, probes on the sex chromosomes were also filtered out (*n* = 11361). The *minfi* preprocessed data were analyzed with *CpGassoc* package to assess differences in DNA methylation profiles between the study groups [31]. A linear fixed effects model was applied for each individual CpG site with age, gender and batch included as covariates in the analysis. The false discovery rate (FDR) calculated according to the Benjamini-Hochberg method was set to 0.05. In a secondary set of analyses major depressive disorder (MDD), positive PTSD screen, coronary heart disease, diabetes, length of education and estimated proportion of buccal epithelial cells were also included as covariates. The proportion of buccal epithelial cells versus leukocytes in the buccal swabs was estimated according to a previously published method [32]. Finally, the data were checked for the possible effect of methylation outliers through visually inspecting CpG plots and including the *minfi* function fixMethOutliers (object, K = −3, verbose = FALSE) in the analysis. 2 of the top reported 71 associations from the original analysis were excluded by controlling for outliers (cg16669619 and cg14895646) and they were not mapped to genes included in the downstream gene enrichment analysis, suggesting that methylation outliers did not affect the observed results. For statistical analysis, M-values, which represent log2-ratios of methylated and unmethylated probes intensities and have been shown to have higher statistical validity, were used [33]. The M-value method has been shown to have superior performance in detection rate and true positive rate of both unmethylated and highly methylated CpG sites [33]. For graphic representation of the data, beta values were employed due to their more intuitive visual interpretation.

### Gene enrichment analysis

Genes in the former indentured child labor group showing significant CpG site differential methylation were analyzed for annotation to biological pathways and their overlap using the Database for Annotation, Visualization and Integration Discovery (DAVID) [34], ToppGene Suite [35] and Pathway Studio [36]. The aim of the analysis was to explore potential biological meaning, even in cases of limited statistical power. DAVID database functional annotation clustering was performed for genes with differential methylation between the two groups. Clusters with enrichment score (ES) > 1.5 and *p* < 0.05 were reported, which is more conservative relative to the generally suggested threshold enrichment score of > 1.3 [34]. Genes from the human genome were used as background. In the ToppGene Suite, the ToppFun module was used to analyze functional enrichment in biological processes of differentially methylated genes. Enrichment for genes from input ≥ 3 and FDR ≤ 0.05 after Benjamini-Hochberg correction for multiple testing is reported. In Pathway Studio gene set enrichment analysis was carried out with a cut-off of for genes from input ≥ 3 and *p* < 0.05. No correction was applied for the number of CpG sites genes have measured on the microarray.

## Results

Former child laborers and controls did not differ significantly based on age (mean ± SD 75.9 ± 5.8 years for the former child labor group compared to 72.8 ± 5.8 years for the control group) and gender (14 F/16 M for the former child labor group compared to 8 F/7 M for the control group). There were no significant differences between the two groups in regards to household income, alcohol or substance dependence/abuse, blood pressure, and/or cholesterol levels. PTSD symptoms, coronary heart disease and diabetes were more frequent in the former child labor group, in accordance with the expected higher prevalence of these disorders after childhood trauma. The control group reported more years of education. Positive depressive symptoms screen was more commonly observed in the former child labor group, but the two groups did not differ significantly in the incidence of MDD diagnosis. Smoking status was known for only a subset of the individuals involved in the study (over 90% of both former indentured child laborers and controls for whom the smoking status was known were non-smokers). We assessed the methylation levels of cg05575921 (annotated to the aryl hydrocarbon receptor repressor), whose methylation was recently found to be a sensitive and specific marker for smoking status in adults [37]. Mean DNA methylation levels of cg05575921 did not differ between the two groups (*p*-value 0.61), implying similarity of the smoking status between the two groups as a whole. Medication status was also known only for a subset of the individuals included in the study, but due to the advanced age of the study population over 80% of participants with known status in both groups were taking a medication. Childhood trauma severity scores were significantly higher in the former child labor group in comparison to the control group. Demographic and childhood trauma characteristics of the participants are presented in Table 1 and Additional file 1: Table S1.

**Table 1:** Summary of the demographic and childhood trauma characteristics of the subjects included in the study

	Child laborer	Control	*p*-value
N for analysis	*N* = 30	*N* = 15	
Age (years; mean ± SD)	75.9 ± 5.8	72.8 ± 5.8	0.103
Gender (F/M)	14 F/16 M	8 F/7 M	0.673
CTQ - total (mean ± SD)	82.2 ± 16.2	34.4 ± 7.89	<0.001
CTQ physical abuse	17.3 ± 6	5.7 ± 1.2	
CTQ emotional abuse	17.4 ± 5.2	6.3 ± 1.4	
CTQ sexual abuse	9.5 ± 7	5.4 ± 1.3	
Age at indenture (years; mean ± SD)	4.8 ± 4.6	none	
Duration of indenture (years; mean ± SD)	11.6 ± 5	none	

CTQ = childhood trauma questionnaire; *p*-values represent results from *t*-test for age, Mann-Whitney test for CTQ total score, and chi-square test for gender

### Differential methylation between former indentured child laborers and controls

**Fig. 1:**
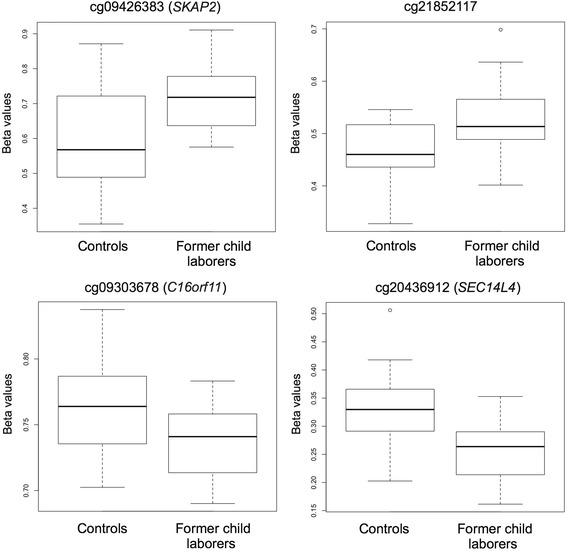
*Box plots* representing the top four differentially methylated positions between the former indentured child labor and control groups

We found 71 differentially methylated CpG positions between the former indentured child laborer and control group, after accounting for age, gender, batch effects, and multiple comparisons. The list of differentially methylated positions meeting experiment-wide significance set at FDR < 0.05 is presented in Table 2, while β-values in the two groups for these positions are presented in Additional file 1: Table S3. One gene – the src kinase associated phosphoprotein 2 (*SKAP2*) - included 5 differentially methylated CpG positions; 41 genes included one differentially methylated position; and 25 differentially methylated positions were located in intergenic regions. Box-plots presenting beta levels between the two groups in the four most significant differentially methylated positions are presented in Fig. 1.

**Table 2:** List of differentially methylated CpG positions between the two groups

CpG number	Chromosome	Gene symbol	*p*-value	FDR	CpG site location within the gene
cg09426383	7	*SKAP2*	1.32 × 10^−7^	0.035	Body
cg21852117	7		3.57 × 10^−7^	0.035	Intergenic
cg09303678	16	*C16orf11*	4.05 × 10^−7^	0.035	TSS1500
cg20436912	22	*SEC14L4*	4.32 × 10^−7^	0.035	TSS1500
cg24377150	1	*SKI*	4.51 × 10^−7^	0.035	Body
cg22718696	1	*MTOR*	4.63 × 10^−7^	0.035	Body
cg02500267	20	*DTD1*	7.45 × 10^−7^	0.045	TSS200
cg19216792	7	*SKAP2*	8.55 × 10^−7^	0.045	Body
cg17256234	11		9.01 × 10^−7^	0.045	Intergenic
cg00325149	1	*COL11A1*	1.11 × 10^−6^	0.047	TSS200
cg12140851	7	*SKAP2*	1.34 × 10^−6^	0.047	Body
cg09997372	17	*CSHL1*	1.65 × 10^−6^	0.047	TSS1500
cg03126199	19	*ZNF486*	1.66 × 10^−6^	0.047	TSS1500
cg26437522	7		1.87 × 10^−6^	0.047	Intergenic
cg14954143	3		1.91 × 10^−6^	0.047	Intergenic
cg12252069	7	*SKAP2*	1.96 × 10^−6^	0.047	Body
cg22519265	17	*ATP2A3*	2.06 × 10^−6^	0.047	5’UTR
cg22324029	6	*HLA-DMB*	2.22 × 10^−6^	0.047	Body
cg14895646	19	*MED25*	2.26 × 10^−6^	0.047	TSS200
cg25121513	7		2.28 × 10^−6^	0.047	Intergenic
cg10993074	6	*C6orf47*	2.37 × 10^−6^	0.047	1^st^ Exon
cg19313023	4		2.44 × 10^−6^	0.047	Intergenic
cg19395289	6		2.53 × 10^−6^	0.047	Intergenic
cg23595621	14	*RNASE6*	2.64 × 10^−6^	0.047	Body
cg18423591	15	*SHF*	2.72 × 10^−6^	0.047	TSS1500
cg10313047	3	*DNAH1*	2.76 × 10^−6^	0.047	5’UTR
cg12074024	1		2.79 × 10^−6^	0.047	Intergenic
cg16669619	18		3.33 × 10^−6^	0.047	Intergenic
cg11923320	1		3.46 × 10^−6^	0.047	Intergenic
cg06793062	16	*CNTNAP4*	3.53 × 10^−6^	0.047	Body
cg11068049	4		3.58 × 10^−6^	0.047	Intergenic
cg07424912	16	*SLC38A7*	3.68 × 10^−6^	0.047	TSS1500
cg00150025	15	*PLA2G4F*	3.74 × 10^−6^	0.047	Body
cg00292312	1	*CAMTA1*	3.79 × 10^−6^	0.047	Body
cg01909551	3		3.88 × 10^−6^	0.047	Intergenic
cg18708013	6	*ZC3H12D*	3.89 × 10^−6^	0.047	5’UTR
cg15641398	7		3.95 × 10^−6^	0.047	Intergenic
cg04910277	8	*DLGAP2*	4.05 × 10^−6^	0.047	Body
cg06226516	7		4.07 × 10^−6^	0.047	Intergenic
cg18431765	2		4.25 × 10^−6^	0.047	Intergenic
cg09168222	4	*HERC6*	4.25 × 10^−6^	0.047	TSS200
cg05941631	2		4.55 × 10^−6^	0.048	Intergenic
cg14889079	19	*NDUFA3*	4.65 × 10^−6^	0.048	TSS200
cg23196756	10	*EIF4EBP2*	4.96 × 10^−6^	0.048	Body
cg21054842	4		5.09 × 10^−6^	0.048	Intergenic
cg00884805	1	*GNG12*	5.13 × 10^−6^	0.048	5’UTR
cg01833485	1	*ESRRG*	5.14 × 10^−6^	0.048	5’UTR
cg01409163	8	*ERICH1*	5.35 × 10^−6^	0.048	Body
cg08101977	16	*CACNA1H*	5.44 × 10^−6^	0.048	Body
cg22026150	8		5.47 × 10^−6^	0.048	Intergenic
cg23385732	14		5.69 × 10^−6^	0.048	Intergenic
cg11891395	7	*DLX5*	5.72 × 10^−6^	0.048	Body
cg24954668	7		5.72 × 10^−6^	0.048	Intergenic
cg22727965	7		5.77 × 10^−6^	0.048	Intergenic
cg24378250	11	*TAF10*	5.88 × 10^−6^	0.049	Body
cg14740251	19		6.32 × 10^−6^	0.049	Intergenic
cg05717473	6		6.52 × 10^−6^	0.049	Intergenic
cg23012579	5	*ERGIC1*	6.65 × 10^−6^	0.049	Body
cg02388718	9	*NCRNA00032*	6.7 × 10^−6^	0.049	Body
cg07936950	2	*DLX1*	6.72 × 10^−6^	0.049	3’UTR
cg00169412	6		6.78 × 10^−6^	0.049	Intergenic
cg03730533	7	*SKAP2*	6.9 × 10^−6^	0.049	Body
cg13432339	4		7.05 × 10^−6^	0.049	Intergenic
cg11782208	1	*C1orf170*	7.15 × 10^−6^	0.049	TSS1500
cg11014810	10	*SH3PXD2A*	7.25 × 10^−6^	0.049	Body
cg11673092	10	*ARMC3*	7.31 × 10^−6^	0.049	TSS1500
cg01573140	7	*OGDH*	7.32 × 10^−6^	0.049	Body
cg02013146	16	*ANKRD11*	7.33 × 10^−6^	0.049	5’UTR
cg19975800	2	*UGT1A10*	7.48 × 10^−6^	0.049	TSS200
cg16270079	3	*PLXNB1*	7.75 × 10^−6^	0.049	Body
cg18703601	3	*ROBO1*	7.82 × 10^−6^	0.049	TSS1500

*P*-values are corrected for batch, gender and age. FDR values have been obtained with the Benjamini-Hochberg method. FDR significance threshold was set at 0.05

*TSS1500* within 1500 bps of a transcription start site, *TSS200* within 200 bps of a transcription start site, *5’UTR* 5’ untranslated region, *3’UTR* 3’ untranslated region

β-values for the CpG positions in both groups are presented in Additional file 1: Table S3

### Enrichment analysis of differentially methylated genes between the former indentured child labor and control groups

Functional annotation clustering of genes with differentially methylated CpG sites using the DAVID database revealed two clusters (Table 3). One of them included genes related to the regulation of cell projection and cellular component organization (ES 1.74, genes: *MTOR; PLXNB1; ROBO1*), while the second involved genes related to neuronal projection/dendrites (ES 1.65, genes: *SH3PXD2A; CACNA1H; DLGAP2; DNAH1; MTOR; ROBO1*). Using the ToppGene Suite, we found enrichment of genes with differentially methylated CpG sites implicated among others in telencephalon development (*ROBO1; DLX1; SKI; GNG12; OGDH*) and olfactory bulb development (*ROBO1; SKI; OGDH*) (Additional file 1: Table S2). Overlap of Pathway Studio analysis data with the other gene enrichment tools included enrichment in genes related to multicellular organismal development (*DLX1; DLX5; ESRRG; PLXNB1; ROBO1*), dendrites (*CACNA1H*; *DLGAP2*; *CNTNAP4*; *MTOR*) and axon guidance (*CACNA1H*; *PLXNB1; ROBO1; DLX5*).

**Table 3:** Functional annotation cluster analysis of genes differentially methylated in the former indentured child labor group versus the control group according to DAVID with *p* < 0.05

Annotation Cluster 1 (Enrichment Score 1.74)		
Term	Genes	*p*-value
Positive regulation of cell projection organization	*MTOR; PLXNB1; ROBO1*	0.0052
Regulation of cell projection organization	*MTOR; PLXNB1; ROBO1*	0.018
Annotation Cluster 2 (Enrichment Score 1.65)		
Cell projection	*SH3PXD2A; CACNA1H; DLGAP2; DNAH1; MTOR; ROBO1*	0.01
Neuron projection	*CACNA1H; DLGAP2; MTOR; ROBO1*	0.028
Dendrite	*CACNA1H; DLGAP2; MTOR*	0.04

### Effect of psychiatric and somatic comorbidities, education duration and proportion of buccal epithelial cells on differentially methylated genes between the groups

Finally, we assessed the role of some major psychiatric (MDD, positive PTSD screen) and somatic (coronary heart disease, diabetes) comorbidities, education and estimated proportion of buccal epithelial cells on the most significant DNA methylation differences between the former child labor and the control group. DNA methylation differences were evaluated between the four most significantly differentially methylated CpG sites in the primary analysis, as well as CpG sites in genes showing consistent enrichment with the applied gene enrichment tools. We included MDD, positive PTSD screen, coronary heart disease, diabetes, education duration and estimated proportion of buccal epithelial cells as covariates. *P*-values for the assessed CpG sites varied between 9.47 × 10^−6^ and 0.00193 (Tables 4 and 5).

**Table 4:** Effect of adjusting for psychiatric and somatic comorbidities, length of education and estimated proportion of buccal epithelial cells on the top four differentially methylated CpG positions between the two groups

CpG number	Chr	Gene symbol	*p*-value before additional correction	*p*-value
cg09426383	7	*SKAP2*	1.32 × 10^−7^	2.61 × 10^−5^
cg21852117	7		3.57 × 10^−7^	6.4 × 10^−5^
cg09303678	16	*C16orf11*	4.05 × 10^−7^	9.47 × 10^−6^
cg20436912	22	*SEC14L4*	4.32 × 10^−7^	1.59 × 10^−4^

**Table 5:** Effect of adjusting for psychiatric and somatic comorbidities, length of education and estimated proportion of buccal epithelial cells on differentially methylated CpG positions between the two groups implicated consistently by the applied gene enrichment tools

CpG number	Chr	Gene symbol	*p*-value before additional correction	*p*-value
cg11891395	7	*DLX5*	5.72 × 10^−6^	0.00159
cg07936950	2	*DLX1*	6.72 × 10^−6^	0.00148
cg01833485	1	*ESRRG*	5.14 × 10^−6^	0.000132
cg18703601	3	*ROBO1*	7.82 × 10^−6^	3.55 × 10^−5^
cg01573140	7	*OGDH*	7.32 × 10^−6^	0.00193
cg08101977	16	*CACNA1H*	5.44 × 10^−6^	0.00139
cg04910277	8	*DLGAP2*	4.05 × 10^−6^	0.000393
cg06793062	16	*CNTNAP4*	3.53 × 10^−6^	0.000423
cg22718696	1	*MTOR*	4.63 × 10^−7^	0.000378
cg16270079	3	*PLXNB1*	7.75 × 10^−6^	0.000549

## Discussion

In the present study we assessed differences in DNA methylation patterns between former indentured child laborers and controls with similar demographic characteristics. Overall, we found DNA methylation differences in 71 CpG positions meeting the experiment-wide significance criteria of FDR < 0.05 between the two groups. Psychiatric and somatic comorbidities and estimated proportion of epithelial cells contribiute partially to the detected differences.

The gene that showed the strongest difference in methylation patterns was *SKAP2,* which included 5 differentially methylated positions. *SKAP2* is an adaptor protein that plays an important role in src signaling and is involved in a wide range of intracellular processes, such as suppression of cell migration and tumor invasion by inhibition of actin polymerization [38–40]. The gene has also been shown to negatively affect the phosphorylation of alpha-synuclein - a protein critically involved in neurodegenerative disorders such as Parkinson’s disease - after cellular stress [41]. Interestingly, the src-kinase for which SKAP2 is an adaptor protein, converges with the WNT pathway recently implicated in DNA methylation alterations in 45 years old individuals subjected to childhood maltreatment [10, 42]. In our study, all of the 5 detected *SKAP2* differentially methylated positions were located in the gene body and all of them showed hypermethylation in the former indentured child labor group. DNA methylation in gene promoters has long been shown to interfere with gene transcription [7]. A role of DNA methylation in gene bodies for gene expression has been suggested, but the exact mechanism and effect are still not conclusively clarified [43, 44]. Since we found methylation changes in CpG sites located both in promotor and gene body regions (Table 1), the effect on gene and protein expression cannot be directly predicted and warrants further investigation.

We further found enrichment of genes with differentially methylated CpG sites in cell/neuronal projection (*CACNA1H*; *DLGAP2*; *MTOR*; *ROBO1; SH3PXD2A*), dendrites (*CACNA1H*; *DLGAP2*; *CNTNAP4*; *MTOR*), axon guidance (*CACNA1H*; *PLXNB1; ROBO1; DLX5*), brain development (*ROBO1; DLX1; SKI; GNG12; OGDH*) and multicellular organismal development (*DLX1; DLX5; ESRRG; PLXNB1; ROBO1*). Our findings are in line with previous studies also reporting links of childhood trauma with genes related to cell signaling and neuronal development [8–11]. In particular, DLG associated protein 2 (*DLGAP2*) and mechanistic target of rapamycin (*MTOR*) have previously been associated with traumatic stress. In a rat model of PTSD increased *DLGAP2* DNA methylation levels and decreased mRNA expression levels have been observed [45]. *MTOR* has been shown to regulate fear memory reconsolidation [46] and its activation in a rat PTSD model has been demonstrated [47]. Our study focused on elderly individuals subjected to childhood trauma, while most of previous investigations have focused on young to middle aged adults [48]. The aging process itself is associated with DNA methylation alterations and traumatic stress can accelerate epigenetic aging [49]. However, it has been shown that some early-life induced DNA methylation changes are protected from erasure associated with age [48]. The extent to which age-associated DNA methylation alterations obliterate the ones induced by early-life trauma in our study population is not possible to predict.

About 30% of the differentially methylated CpG positions detected were located in intergenic regions. A previous study by Yang et al. investigating abused children found about 20% of the differentially methylated positions to be in intergenic regions [11]. Changes in DNA methylation in intergenic regions may play an important role in the genome organization and gene expression regulation through the binding sites for transcription factors located in them [50].

Our study has several limitations. These include the relatively small sample size and the cross-sectional design of the study. Due to the old age of the participants, comorbidities and medication intake were very common, making controlling for potential confounding factors difficult. Even though we assessed the role of certain psychiatric and somatic comorbidities, additional controls and replication in larger study cohorts are needed. In addition, we investigated a unique group of individuals subjected to prolonged and severe traumatic experience in their childhood, and it may not be possible to extrapolate detected differences directly to other study populations. Finally, we used a peripheral tissue – buccal epithelial cells for detection of DNA methylation patterns. While epigenetic patterns are tissue-specific, DNA methylation alterations in response to early life adversity have been shown to be a system-wide phenomenon, and studying peripheral tissues may yield important clues to the biological pathways alterations induced [51].

## Conclusions

Altogether, we found differences in DNA methylation profiles of elderly individuals subjected to prolonged and severe childhood trauma in comparison to controls with similar demographic characteristics. They encompass an adaptor protein for the src-kinase, genes related to development, cellular and neuronal projection. Other psychiatric and somatic comorbidities and estimated proportion of buccal epithelial cells appear to partially contribute to the here observed DNA methylation differences.

## Additional file

Additional file 1: Supplementary data. (DOCX 29 kb)

## Abbreviations

3’UTR: 3’ untranslated region
5’UTR: 5’ untranslated region
CTQ-SF: Childhood trauma questionnaire – short form
DAVID: Database for annotation, visualization and integration discovery
DLGAP2: DLG associated protein 2
ES: Enrichment score
F: Female
FDR: False discovery rate
IDAT file: Raw intensity data file
M: Male
MDD: Major depressive disorder
MTOR: Mechanistic target of rapamycin
PTSD: Posttraumatic stress disorder
SD: Standard deviation
SKAP2: Src kinase associated phosphoprotein 2
SNP: Single nucleotide polymorphism
TSS1500: Within 1500 bps of a transcription start site
TSS200: Within 200 bps of a transcription start site
