# Supplementary material for: DNA methylation profiles of elderly individuals subjected to indentured childhood labor and trauma
Source: BMC Med Genet. 2017 Feb 27;18:21. doi: 10.1186/s12881-017-0370-2 (PMC5329963; doi:10.1186/s12881-017-0370-2)
Supplement: Additional file 1: — Supplementary data. (DOCX 29 kb) [file 12881_2017_370_MOESM1_ESM.docx]

**Supplementary Methods**

*Psychometric assessments of the study groups*

All participants in the study were screened for alcohol dependence/alcohol abuse and substance dependence/substance abuse with the relevant questions of the Mini International Neuropsychiatric Interview (M.I.N.I.) [1]. Depressive symptoms were evaluated with the Geriatric Depression Scale (GDS) - a 15-item self-report questionnaire for the assessment of clinical depression in the elderly, which has demonstrated high reliability (α=0.81), while major depressive disorder was asked about with M.I.N.I. [1, 2, 3]. For a positive depression screen, participants had to report depression symptoms in at least 5 of the questions. A cut-off value of 5 in the GDS has previously demonstrated 71.8% sensitivity and 78.2% specificity [4].

Former indentured child laborers and controls were questioned about the three most severe traumatic experiences in their adult lifespan. The trauma events list of the Munich-Composite International Diagnostic Interview (M-CIDI; German version: Diagnostisches Expertensystem für psychische Störungen: DIA-X) was used to register such potential traumatic events [5]. The DIA-X is an extended version of the CIDI [6], which addressed the need to improve compatibility with DSM-IV diagnoses and made some methodological changes to the original version. The trauma list of DIA-X lists major potential traumatic events to the occurrence of which the interviewed person answers with “yes” or “no”. The M-CIDI has demonstrated high test-retest reliability for most diagnostic entities assessed, including excellent test-retest reliability for posttraumatic stress disorder (kappa=0.79) [7]. PTSD symptoms were assessed with the short screening scale (SSS) for DSM-IV PTSD, which consists of 7 items [8]. 5 of the items assess symptoms from the avoidance and numbing cluster C, while two of the items assess symptoms from the hyperarousal cluster D. A German version of the SSS scale is available and was used in the current study [9]. For positive PTSD screen at least 4 of the 7 symptoms had to occur two times or more per week, corresponding to the original design of the scale. The SSS scale was assessed for each of the three most severe traumatic events in the lives of the interviewed individuals.

Prevalence of diabetes, elevated cholesterol levels, elevated blood pressure or coronary artery disease, length of education and household income were assessed by targeted questions.

**Supplementary references:**

1. Sheehan DV, Lecrubier Y, Sheehan KH, Amorim P, Janavs J, Weiller E, Hergueta T, Baker R, Dunbar GC. The Mini-International Neuropsychiatric Interview (M.I.N.I.): the development and validation of a structured diagnostic psychiatric interview for DSM-IV and ICD-10*.* The Journal of Clinical Psychiatry. 1998;59:22-33.
2. Sheikh JI, Yesavage JA. Geriatric Depression Scale (GDS): Recent evidence and development of a shorter version. Clinical Gerontologist: The Journal of Aging and Mental Health. 1986;5:165–73.
3. Wancata J, Alexandrowicz R, Marquart B, Weiss M, Friedrich F. The criterion validity of the Geriatric Depression Scale: a systematic review. Acta Psychiatrica Scandinavica 2006;114:398-410.
4. Marc LG, Raue PJ, Bruce ML. Screening performance of the 15-item geriatric depression scale in a diverse elderly home care population. The American Journal of Geriatric Psychiatry. 2008;16:914-21.
5. Wittchen HU, Pfister H. Diagnostisches Expertensystem für psychische Störungen (DIA-X). Frankfurt a.M.: Swets&Zeitlinger. 1997.
6. Wittchen HU. Reliability and validity studies of the WHO--Composite International Diagnostic Interview (CIDI): a critical review. Journal of Psychiatric Research. 1994;28:57–84.
7. Wittchen HU, Lachner G, Wunderlich U, Pfister H. Test-retest reliability of the computerized DSM-IV version of the Munich-Composite International Diagnostic Interview (M-CIDI). Social Psychiatry and Psychiatric Epidemiology 1998;33:568-78.
8. Breslau N, Peterson EL, Kessler RC, Schultz LR. Short screening scale for DSM-IV posttraumatic stress disorder. The American Journal of Psychiatry. 1999;156:908-11.
9. Siegrist P, Maercker A. Deutsche Fassung der Short Screening Scale for DSM-IV Posttraumatic Stress Disorder. Aktueller Stand der Validierung. Trauma & Gewalt. 2010;4:208–13.

**Supplementary Table S1.** Further demographic and comorbidity data on the study participants**.**

|  | **Child laborer** | **Control** |
| --- | --- | --- |
| Alcohol dependence | N = 1 (3.3%) | N = 0 (0%) |
| Substance dependence | N = 1 (3.3%) | N = 0 (0%) |
| PTSD positive screen | N = 8 (26.7%) | N = 0 (0%) |
| GDS positive screen | N = 9 (30%) | N = 0 (0%) |
| Major depressive disorder | N = 2 (6.6%) | N = 0 (0%) |
| Elevated cholesterol | N =13 (43.3%) | N = 5 (33.3%) |
| Elevated blood pressure | N = 17 (56.7%) | N = 6 (40%) |
| Coronary artery disease | N = 8 (26.7%) | N = 0 (0%) |
| Diabetes | N = 5 (16.7%) | N = 1 (6.7%) |
| Education (in years) | 9.9 ± 2.8 | 14.1 ± 3.7 |
| Current household income  (>50’000 /<50’000 per year) | 17 / 12  (1 unknown) | 9 / 6 |

*Note:* PTSD = posttraumatic stress disorder; GDS = geriatric depression scale

**Supplementary Table S2.** Enrichment of differentially methylated genes in the former indentured child labor group compared to the control group in biological processes according to ToppGene with FDR < 0.05.

| **ID** | **Name** | **p-value** | **FDR** |
| --- | --- | --- | --- |
| GO:0021772 | olfactory bulb development | 0.000068 | 0.0421 |
| GO:0060325 | face morphogenesis | 0.000074 | 0.0421 |
| GO:0021988 | olfactory lobe development | 0.000080 | 0.0421 |
| GO:0060323 | head morphogenesis | 0.000134 | 0.045 |
| GO:0021537 | telencephalon development | 0.000199 | 0.0421 |
| GO:0060322 | head development | 0.000214 | 0.0486 |
| GO:0060324 | face development | 0.000255 | 0.0495 |
| GO:0010171 | body morphogenesis | 0.000280 | 0.0495 |

**Supplementary Table S3.** Mean β levels for the two groups and ∆β between the groups

| **CpG number** | **β controls** | **β child laborers** | **∆β** |
| --- | --- | --- | --- |
| cg09426383 | 0.572 | 0.71 | -0.138 |
| cg21852117 | 0.456 | 0.522 | -0.067 |
| cg09303678 | 0.813 | 0.804 | 0.0086 |
| cg20436912 | 0.335 | 0.261 | 0.074 |
| cg24377150 | 0.507 | 0.418 | 0.089 |
| cg22718696 | 0.501 | 0.455 | 0.045 |
| cg02500267 | 0.0475 | 0.0461 | 0.0014 |
| cg19216792 | 0.613 | 0.714 | -0.101 |
| cg17256234 | 0.774 | 0.722 | 0.051 |
| cg00325149 | 0.3376 | 0.3969 | -0.059 |
| cg12140851 | 0.671 | 0.8145 | -0.144 |
| cg09997372 | 0.557 | 0.509 | 0.048 |
| cg03126199 | 0.7497 | 0.7302 | 0.0195 |
| cg26437522 | 0.361 | 0.426 | -0.065 |
| cg14954143 | 0.549 | 0.599 | -0.05 |
| cg12252069 | 0.685 | 0.812 | -0.127 |
| cg22519265 | 0.437 | 0.409 | 0.028 |
| cg22324029 | 0.485 | 0.538 | -0.05 |
| cg14895646 | 0.034 | 0.019 | 0.015 |
| cg25121513 | 0.432 | 0.499 | -0.067 |
| cg10993074 | 0.537 | 0.462 | 0.075 |
| cg19313023 | 0.4 | 0.343 | 0.057 |
| cg19395289 | 0.437 | 0.409 | 0.028 |
| cg23595621 | 0.505 | 0.559 | -0.054 |
| cg18423591 | 0.522 | 0.453 | 0.069 |
| cg10313047 | 0.792 | 0.859 | -0.067 |
| cg12074024 | 0.369 | 0.26 | 0.11 |
| cg16669619 | 0.904 | 0.851 | 0.053 |
| cg11923320 | 0.357 | 0.401 | -0.044 |
| cg06793062 | 0.453 | 0.332 | 0.121 |
| cg11068049 | 0.8857 | 0.8843 | 0.0013 |
| cg07424912 | 0.713 | 0.679 | 0.034 |
| cg00150025 | 0.361 | 0.214 | 0.147 |
| cg00292312 | 0.528 | 0.459 | 0.07 |
| cg01909551 | 0.691 | 0.61 | 0.082 |
| cg18708013 | 0.422 | 0.474 | -0.051 |
| cg15641398 | 0.415 | 0.365 | 0.051 |
| cg04910277 | 0.466 | 0.402 | 0.064 |
| cg06226516 | 0.763 | 0.761 | 0.002 |
| cg18431765 | 0.726 | 0.622 | 0.104 |
| cg09168222 | 0.418 | 0.36 | 0.058 |
| cg05941631 | 0.343 | 0.373 | -0.03 |
| cg14889079 | 0.068 | 0.037 | 0.031 |
| cg23196756 | 0.215 | 0.171 | 0.045 |
| cg21054842 | 0.588 | 0.642 | -0.05 |
| cg00884805 | 0.486 | 0.404 | 0.082 |
| cg01833485 | 0.713 | 0.756 | -0.043 |
| cg01409163 | 0.74 | 0.716 | 0.023 |
| cg08101977 | 0.402 | 0.367 | 0.035 |
| cg22026150 | 0.527 | 0.472 | 0.055 |
| cg23385732 | 0.302 | 0.239 | 0.063 |
| cg11891395 | 0.505 | 0.634 | -0.129 |
| cg24954668 | 0.256 | 0.33 | -0.074 |
| cg22727965 | 0.379 | 0.44 | -0.062 |
| cg24378250 | 0.529 | 0.477 | 0.052 |
| cg14740251 | 0.64 | 0.724 | -0.084 |
| cg05717473 | 0.667 | 0.606 | 0.061 |
| cg23012579 | 0.054 | 0.035 | 0.0195 |
| cg02388718 | 0.763 | 0.743 | 0.02 |
| cg07936950 | 0.326 | 0.401 | -0.075 |
| cg00169412 | 0.147 | 0.18 | -0.033 |
| cg03730533 | 0.545 | 0.686 | -0.141 |
| cg13432339 | 0.323 | 0.348 | -0.025 |
| cg11782208 | 0.576 | 0.515 | 0.061 |
| cg11014810 | 0.738 | 0.715 | 0.023 |
| cg11673092 | 0.345 | 0.4 | -0.055 |
| cg01573140 | 0.852 | 0.894 | -0.042 |
| cg02013146 | 0.9064 | 0.9001 | 0.006 |
| cg19975800 | 0.435 | 0.354 | 0.081 |
| cg16270079 | 0.702 | 0.589 | 0.113 |
| cg18703601 | 0.267 | 0.321 | -0.054 |

*Note:* ∆β represents the result from mean β values for the control group minus mean β values for the former child labor group (not corrected).
